# Note from Dr. Wallace

**Published:** 1848-11

**Authors:** Ellerslie Wallace

**Affiliations:** 309 Walnut street.; Philadelphia


### *Note from Dr. Wallace.*

To the Editor of the Medical Examiner.

Dear Sir,—In the number of the Examiner published July, 1847, you gave place to a report of "Extirpation of the Parotid Gland, by Professor Pancoast." The report was not complete, inasmuch as the patient left this city previous to the separation of the ligature applied to the external carotid artery. I was for a long time unable to obtain any information with regard to her, but have now the pleasure to inform you, that the ligature came away about a fortnight after she reached her home, and from that time to the present she has enjoyed uninterrupted health. I furthermore learn that there is no appearance of any return of the disease, for the relief of which the operation was performed.

Very respectfully,

Ellerslie Wallace,

309 Walnut street.

*Philadelphia, Sept.* 20*th*, 1848.

